# Influence of Ethylene Oxide Content in Nonionic Surfactant to the Hydrolysis of Reactive Dye in Silicone Non-Aqueous Dyeing System

**DOI:** 10.3390/polym10101158

**Published:** 2018-10-17

**Authors:** Jiping Wang, Yongbo Zhang, Huashu Dou, Liujun Pei

**Affiliations:** 1 Engineering Research Center for Eco-Dyeing and Finishing of Textiles, Zhejiang Sci-Tech University, Hangzhou 310018, China; jpwang@zstu.edu.cn (J.W.); 15990048685@163.com (Y.Z.); 2 National Base for International Science & Technology Cooperation in Textiles and Consumer-Goods Chemistry, Zhejiang Sci-Tech University, Hangzhou 310018, China; 3 Key Laboratory of Fluid Transmission Technology of Zhejiang Province, Zhejiang Sci-Tech University, Hangzhou 310018, China; huashudou@zstu.edu.cn

**Keywords:** hydrolysis, dye-surfactant interaction, siloxane non-aqueous reverse emulsion dyeing, EO chains, vinyl sulfone reactive dye

## Abstract

Silicone reverse dyeing technology provides an important means of saving water and salts-free in the textile dyeing industry. The interactions between dyes and surfactants may influence the hydrolysis of dye during dyeing. In this investigation, the effect of ethylene oxide content in nonionic surfactant on the hydrolytic reaction of reactive dye was firstly investigated in a siloxane reverse emulsion dyeing system. Compared with no surfactants, the hydrolytic reaction of vinyl sulfone reactive dye was a slowdown when some nonionic surfactants were used during dyeing. Usually, the hydrophobic groups in nonionic surfactants were dodecyl chains but their polar head groups were different. The hydrolytic reaction of vinyl sulfone dye showed that the longer of EO (ethylene oxide) chains, the faster the hydrolytic reaction of vinyl sulfone dye. From the absorption spectrum of dye, it could be concluded that more of dyes would be solubilized into the formed micelles, and dye-surfactant complexes were adhered to the surface of micelles if the molecular structure of surfactant had a shorter EO chains. Furthermore, the intramolecular or intermolecular hydrogen bond could be formed between dye and surfactant, which would further influence the hydrolytic reaction of vinyl sulfone dye. However, the solubility of surfactant in siloxane non-aqueous media would decrease with the increase of EO chains. Meanwhile, the dispersion of dye was enhanced as well as the hydrolytic reaction of dye. From this investigation, some surfactant can be used to improve the fixation of reactive dye during dyeing. Furthermore, washing times after dyeing and the ecological problems can be decreased.

## 1. Introduction

The interactions between dyes and surfactants are important in various industry processes such as printing ink, textile dyeing and inhabitation of dye transfer in detergency as well as chemical research on areas such as photosensitization, analytical chemistry and biochemistry [1,2,3]. The amount of surfactant is also added into the dyeing bath which used as refining agent, leveling agent, dispersant, soaping agent, etc. [4,5]. Generally, the presence of surfactant improves the dyeing properties, especially for the leveling effect of dye. As is well known, the fixation process is also quite important for reactive dyeing. Hence, increasing the fixation and reducing the hydrolysis of reactive dye are important for reactive dyeing.

In our previous investigations, siloxane non-aqueous media were chosen as continuous phase media to prepare siloxane reverse emulsion system or suspension system for dyeing cotton textile with reactive dye [6,7]. With the aid of surfactant and co-surfactant, the dye solution could be evenly emulsified in siloxane non-aqueous medium. Since reactive dye was totally incompatible with siloxane, there was a strong affinity between the fiber and the dye, with the result that nearly 100% of reactive dye can diffuse to the surface of cotton textile under mechanical force [7]. Furthermore, the dyeing temperature was low (25~30 °C) and no inorganic salts were added as an accelerating agent during dyeing. Since only a small quantity water was used in the process, the hydrolysis rate of reactive dye was slower than that in a conventional aqueous solution dyeing system, as well as the color depth of the dyed fabric being much better in siloxane base dyeing. The reasons for the slower hydrolysis of reactive dye were mainly because the grasping ability of surfactant for dye aqueous solution, the faster dyeing rate, the lower dyeing temperature (30 °C in siloxane non-aqueous dyeing system vs. 60 °C in conventional aqueous solution dyeing system), etc. [8,9,10]. When comparing the hydrolysis of dye in siloxane non-aqueous dyeing system, the hydrolytic reaction of dye was faster when surfactant was not added during dyeing. Therefore, the interaction of dye and surfactant might influence the hydrolysis of reactive dye in a siloxane non-aqueous dyeing system [11].

As is known to all, the interactions between dyes and surfactants can be investigated with spectrophotometry [11,12,13], potentiometry [14,15], voltammetry [16] and conductometry [17] methods. Among them, the spectrophotometric method is very suitable. Tunc S. [18] investigated the interactions between dyes and various surfactants with spectrophotometric method in an aqueous solution, and results showed that the solubility of dye was enhanced due to micelle formation. Meanwhile, dye was solubilized primarily as the monomer micelle at a concentration below critical micelle concentration (CMC). If the concentration of surfactant was below CMC, surfactant mainly serves as a dispersant [19]. Chen K. [20] studied the interactions between multi-anionic surfactants and direct dyes with spectrophotometric method, and the surfactants effects on the dyeing of cotton fabrics were also investigated. In the presence of the surfactants, the rate of direct dyeing for cotton fabric depended on the extent of dye disaggregation or dye-surfactant complexation in the dyeing bath. Once the concentration of surfactant was increased to near or above CMC, some of dyes were dissolved into the micellar phase and the absorbances intensity of dye was increased. Oakes J. [4,5] systematically investigated the solubilization of azo dyes by different surfactants including nonionic, anionic, cationic and zwitterionic surfactant. The presence of surfactant did change the environment of dye during dyeing, and the attractive or repulsive electrostatic interactions played a key role in dye binding to micelles.

Nonionic surfactants are typically added as an emulsifier in a siloxane non-aqueous dyeing system [7,8]. However, information about the influence of nonionic surfactants on the hydrolytic behavior of reactive dye is limited in the siloxane non-aqueous emulsion dyeing system; in particular, the detailed information about the effect of the ethylene oxide content on the hydrolytic behavior of reactive dye has not been systematically studied.

In this investigation, some nonionic surfactants with different ethylene oxide content were used to prepare a siloxane reverse emulsion dyeing system. Reactive blue 19 was chosen to as a model of reactive dye and a popular nonionic surfactant alkyl alcohol polyoxyethylene ether which contains a different EO chain was selected as a model of nonionic surfactant. The hydrolytic reaction of blue 19 was studied using HPLC (high performance liquid chromatography) method. The interaction between reactive blue 19 and alkyl alcohol polyoxyethylene ether was investigated using the UV-spectrophotometry method.

## 2. Experimental

### 2.1. Materials

**Figure 1:**
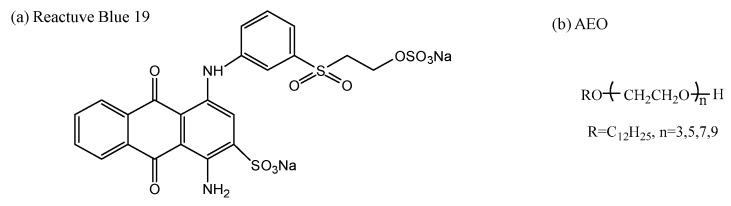
Structure of reactive blue 19 and AEO.

C.I. Reactive Blue 19, acetonitrile, tetrabutylammonium bromide, and ammoniumacetate (chromatographic grade) were obtained from Aladdin Reagent (Shanghai, China). Structure of vinyl sulfone reactive dye is shown in Figure 1a. Siloxane non-aqueous medium with purity above 98% was purchased from Wynca (Jiande, China). Alkyl alcohol polyoxyethylene ether with different number of EO (3, 5, 7 and 9) was purchased from Jiangsu Haian Petrochemical Plant (Haian, China). Structure of AEO is shown in Figure 1b. Acetic acid, sodium carbonate, sodium acetate, sodium bicarbonate and *n*-octanol were purchased from Cangzhou Haolong Chemical Products Co., Ltd (Cangzhou, China).

### 2.2. UV Analysis

The concentration of reactive blue 19 was kept constant at 0.15 mmol/L in this investigation and the absorption spectra in different concentration of AEO-3 were recorded using a Lambda 35 UV-Vis spectrophotometer (Perkin Elmer, Waltham, MA, USA). Then the surfactants solutions of fatty alcohol polyoxyethylene ether (C_12_–C_13_ carbon chain) which contain different content of ethylene oxide (AEO-3, AEO-5, AEO-7, AEO-9) at a concentration of 0.168 mmol/L (below CMC) and 4.60 mmol/L (above CMC) were prepared to study the effect of EO on UV absorption spectra of vinyl sulfone dye.

### 2.3. Preparation of the Buffer Solution

Acidic buffer solution and alkaline buffer solution were prepared using the method which was introduced in the previous investigations [8,9].

### 2.4. HPLC Analysis

The hydrolytic reaction of reactive blue 19 was identified using HPLC analyzing method (Agilent 1260 Infinity, Palo Alto, CA, USA). Reactive dye solution samples were collected at different time intervals. The hydrolysis of different samples was performed in alkaline buffer solution (pH = 11) and analyzed in acidic buffer solution (pH = 4). Agilent 1260 Infinity was equipped with a C-18 reversed-phase chromatographic column (4.6 × 150 mm) and 3.5 μm film thickness. The temperature of column was set at 30 °C during testing. The samples were filtered with 0.22 mm membrane filter before testing. Mobile phase was referred to the procedure reported in the literature [8]. Gradient elution system was shown in Table 1. The injection volume of samples was 20 μL and the flow rate was 1 mL/min. Dye solutions were detected at 585 nm (maximum absorption wavelength of reactive blue 19).

### 2.5. Hydrolysis of Reactive Blue 19

The concentration of dye was 20 g/L in aqueous solution which was calculated by the siloxane reverse emulsion dyeing system. Therefore, 0.1 g reactive blue 19 was dissolved into 5 mL of the above alkaline buffer solution. 4.60 mmol/L nonionic surfactant was added in the siloxane non-aqueous media, and the above dye solution was emulsified in the mixed solution of siloxane and surfactant. Hydrolytic reaction of dye was stirred at 70 °C, and 5 mL of solution was extracted at various time intervals, cooled, neutralized using the acidic buffer solution and diluted to 25 mL.

## 3. Results and Discussion

### 3.1. Hydrolytic Behavior of Reactive Blue 19 in the Siloxane Son-aqueous Dyeing System

**Figure 2:**
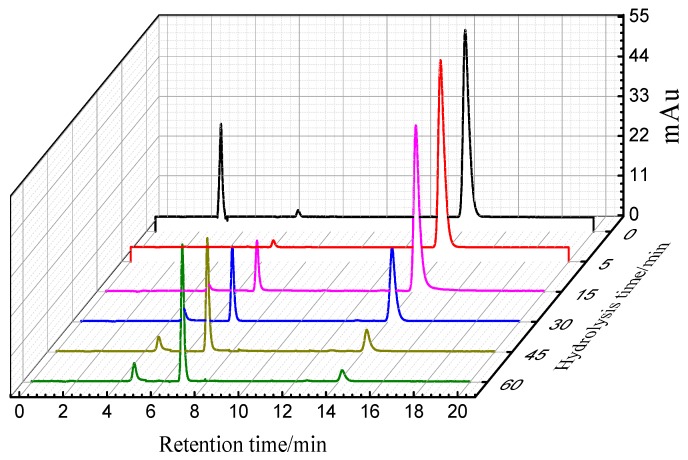
HPLC chromatograms of C.I. Reactive Blue 19 at pH = 11, temperature at 70 °C in siloxane emulsion dyeing system.

Figure 2 shows the hydrolytic behaviors of reactive blue 19 at different hydrolysis times in a siloxane reverse emulsion dyeing system (pH = 11, temperature = 70 °C). The liquid chromatograms showed that four peaks were detected during the hydrolysis of reactive blue 19. Briefly analyzing, one peak was observed at around 3 min, and disappeared rapidly in an alkaline buffer solution. The area and height of peak at 14.19 min was strong but decreased as the hydrolysis time progressed. For the peaks at 6.92 and 4.63 min, their peak height and area increased with the hydrolytic time.

**Figure 3:**
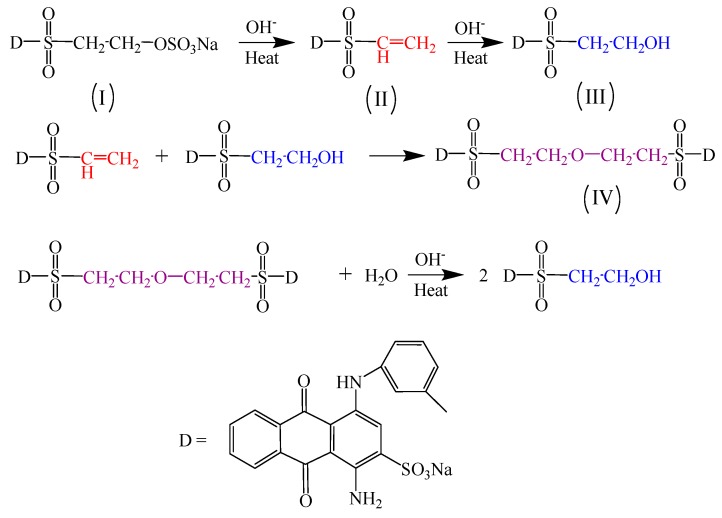
Hydrolytic route of C.I. Reactive Blue 19.

According to Shao J. and Heyna J. [21,22], the hydrolytic route of reactive blue 19 is shown in Figure 3. Commercial reactive blue 19 was stored under a stable state of *β*-sulfatoethylsulfone non-reactive form (I), which can change to vinyl sulfone dye (II) in a short time in an alkaline environment. Furthermore, reactive vinyl sulfone dye added into water can hydrolyze to *β*-hydroxyethyl sulfone dye (III) under an alkaline condition. In additional, reactive vinyl sulfone dye can react with *β*-hydroxyethyl sulfone dye and generate ether dye (IV). Finally, hot strong alkali condition can promote the cleavage of ether dyes to yield *β*-hydroxyethyl sulfone dye. In this work, the impact of ethylene oxide content in nonionic surfactants structure on the hydrolytic property of reactive blue 19 was further deeply investigated using HPLC measurement.

### 3.2. Effect of Ethylene Oxide Content on the Hydrolysis of Vinyl Sulfone Reactive Dye

**Figure 4:**
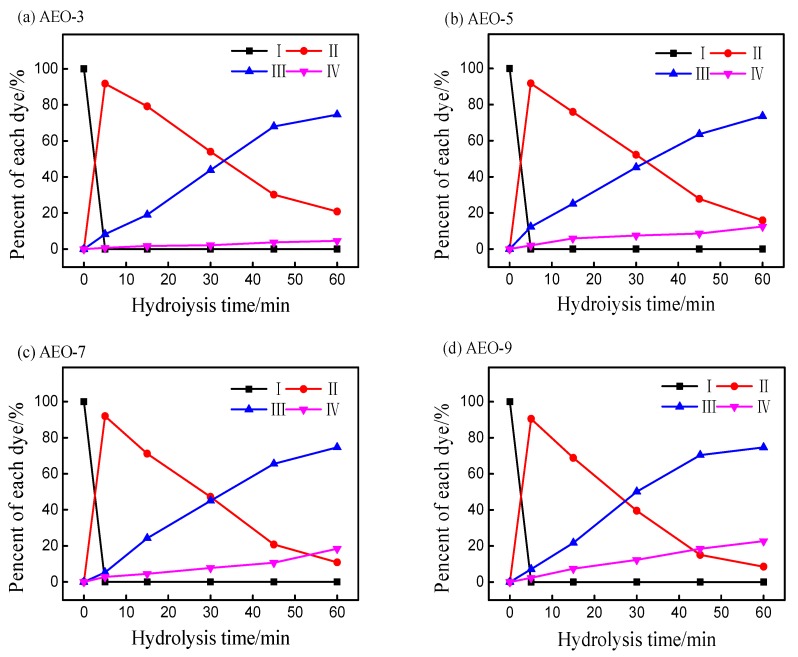
Percent of each peak’s area with the hydrolysis time in siloxane dyeing system at 70 °C: (**a**) AEO-3, (**b**) AEO-5, (**c**) AEO-7, (**d**) AEO-9, and the concentration of surfactants were 4.60 mmol/L.

In order to study the influence of ethylene oxide content in nonionic surfactant on the hydrolysis of dye, different siloxane dyeing systems were prepared and their influence on the hydrolytic reaction of reactive blue 19 was studied. From the Figure 4, it can be concluded that the alkyl alcohol polyoxyethylene ether with different EO chain did affect the hydrolytic behavior of reactive blue 19. First of all, when the surfactant concentration was 4.60 mmol/L (above CMC), the conversion from *β*-sulfato-ethylsulfone dye to vinyl sulfone dye was completed in a short time (less than 5 min) at 70 °C in a siloxane non-aqueous dyeing system. Meanwhile, nonionic surfactant with a long EO chain facilitated the conversion from vinyl sulfone dye to hydrolyzed dye, which was compared with hydrolysis of dye with a shorter EO chain. For example, 20.82% of vinyl sulfone dye was existed after 60 min in siloxane dyeing system, in which AEO-3 was added dyeing formulation (Figure 4a). When AEO-9 was added in siloxane dyeing system, only 8.61% (Figure 4d) of vinyl sulfone dye existed at the same hydrolysis time.

**Figure 5:**
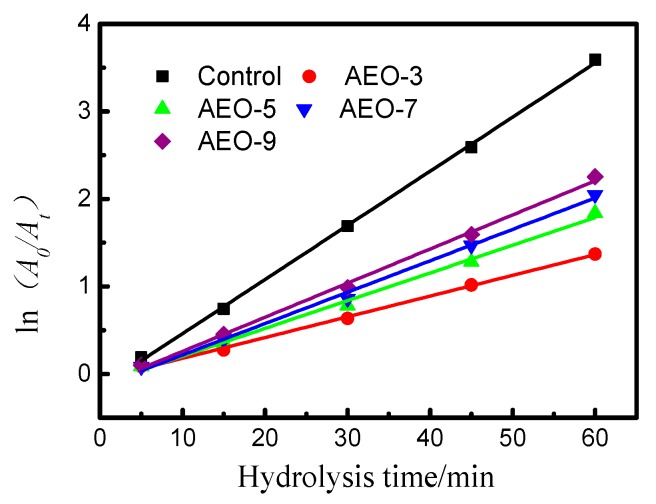
Plot of ln(*A*_o_*/A*_t_) verses time at various ethylene oxide content in siloxane non-aqueous dyeing system.

The hydrolysis of reactive dye is assorted with a nucleophilic addition reaction between dye and hydroxide ions (–OH) [23,24,25]. By assuming that the rate of nucleophilic addition is a constant of the hydrolysis and the concentration of nucelophilic species (–OH) are constant when using a large excess of the alkali buffer solution. The reaction rate of dye at constant temperature can be determined by the following Equation:

−*d*[*C*]/*d*_t_ = *k*[*C*] (1)

where [*C*] refers the concentration of vinyl sulfone dye at reaction time *t* and *k* is the rate constant for hydrolysis. Integrating Equation (1) and replacing the concentrations of the vinyl sulfone dye with the peak areas obtained from the chromatograms can get the following Equation:

ln(*A*_0_/*A*_t_) = *k*_t_ (2)

where *A*_0_ and *A*_t_ refer the peak areas of the active vinyl sulfone dye at *t* = 5 min and the subsequent reaction time *t* in this investigation. A straight line can be obtained by plotting ln(*A*_0_/*A*) against reaction time at constant temperature. Using the Equation (2), pseudo-first-order kinetics can be observed at 70 °C within 60 min (Figure 5). The slope of the line corresponds to the hydrolysis rate constant (*k*).

The overall rate constant, as well as the squares of correlation coefficients in the linear region of the plots are given in Table 2. It was clearly determined that the EO chain in alkyl alcohol polyoxyethylene ether did affect the hydrolytic behavior of dye. When no nonionic surfactants were added to the siloxane non-aqueous reverse emulsion dyeing, the hydrolysis rate constant of blue 19 was 5.47 × 10^−2^/min. However, the hydrolysis rate constant was decreased when AEO-3 was added to the dyeing system. When the number of EO was increased to 9, the hydrolysis rate constant of blue 19 was increased from 2.24 × 10^−2^ to 3.60 × 10 ^−2^/min. Therefore, the greater the number of hydrophilic groups in molecular structure of surfactant, the faster the hydrolysis rate constant of the vinyl sulfone dye.

As the number of hydrophilic groups was low, vinyl sulfone dye solution could be emulsified into siloxane non-aqueous media. Therefore, reactive dyes were solubilized into the formed micelles and were protected from nucleophilic ions [26,27], thus the hydrolytic reaction of dye could be slowed down. However, alkyl alcohol polyoxyethylene ether might play as a dispersant with a long EO chain [28], with the result that reactive dye could not emulsify but was dispersed in the siloxane non-aqueous dyeing system [29]. As a result, hydroxide ions could reach every single dye molecule, which catalytically accelerated the hydrolytic reaction of dye.

### 3.3. Effect of Nonionic Surfactants on UV Spectrum of Vinyl Sulfone Reactive Dye

**Figure 6:**
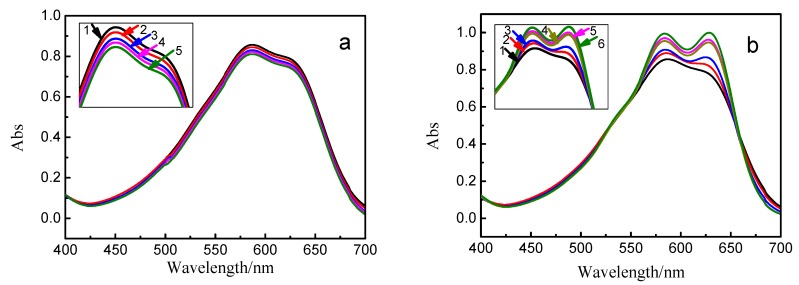
UV-vis spectra of reactive blue 19 (0.16 mmol/L) solution in the presence of AEO-3 with different concentrations. (**a**) [AEO-3] = (1) 0, (2) 0.014, (3) 0.027, (4) 0.045 and (5) 0.084 mmol/L. (**b**) [AEO-3] = (1) 0, (2) 0.14, (3) 0.56, (4) 1.60, (5) 4.60, and (6) 8.93 mmol/L. (Insert images were the enlarge part of the peak area of spectra).

UV-spectrophotometry method was used to study the effect of nonionic surfactants on UV spectrum of vinyl sulfone reactive dye. The molecular weight of AEO-3 used in this work was approximately 317 and the approximate CMC measured by surface tension was about 0.084 mmol/L. As shown in Figure 6, the absorption spectrum of reactive blue 19 showed the maximum absorbance intensity at 585 nm. While in AEO-3 solution (below 0.084 mmol/L), the maximum absorbance intensity decreased slightly with the increasing of AEO-3 concentration (Figure 6a). However, the maximum absorbance intensity of reactive blue 19 increased and a double-peak was formed, which its absorbance intensity increased quickly (Figure 6b) with the increasing of AEO-3 concentration (above 0.084 mmol/L). Therefore, the presence of nonionic surfactant did affect the absorption behavior of reactive dye.

**Figure 7:**
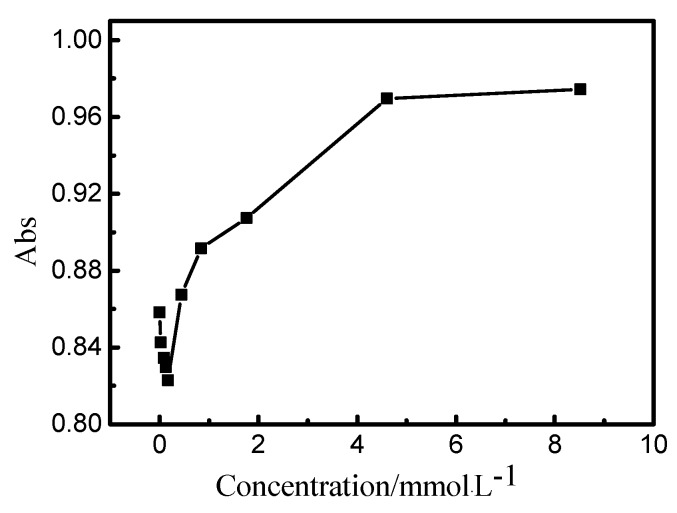
The absorbance intensity of reactive blue 19 with different AEO-3 concentration.

The variation of absorbance intensity of reactive blue 19 with the concentration of AEO-3 was presented in Figure 7. Adsorption peak at 585 nm was used because the monomer form absorbance wavelength at 585 nm for reactive blue 19. Results showed that the monomer absorbance at 585 nm decreased when the concentration of surfactant was below CMC. This mainly because that AEO-3 served as dispersant and a spot of dye-surfactant aggregations formed in submicellar solution [30,31,32,33]. When the concentration of AEO-3 increased to near or above CMC, micelles formed and dye-surfactant aggregations adhered to the micelles surface. The hydrophobic chromophores in the molecular structures of reactive dyes were aggregated near the surface, which would result in more efficient light absorption and an increase in absorbance intensity.

### 3.4. Effect of the EO Chains on UV Spectrum of Vinyl Sulfone Reactive Dye

**Figure 8:**
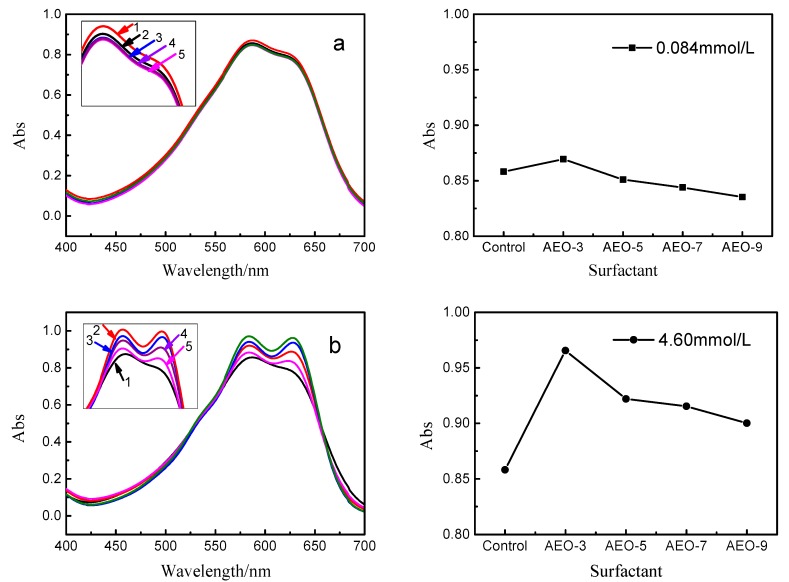
Absorption spectra of reactive blue 19 (0.16 mmol/L) in the presence of alkyl alcohol polyoxyethylene ether with different poly-EO chains. (**a**) [concentration = 0.084 mmol/L]: (1) AEO-3, (2) control, (3) AEO-5, (4) AEO-7, (5) AEO-9; (**b**) [concentration = 4.60 mmol/L]: (1) control, (2) AEO-3, (3) AEO-5, (4) AEO-7, (5) AEO-9.

From the above research, the EO chains in molecular structure of alkyl alcohol polyoxyethylene ether did affect the hydrolysis of vinyl sulfone dye. This phenomenon may be attributed to the change of micro-environment of the reactive dye. As shown in Figure 8a, when the nonionic surfactant concentration was 0.084 mmol/L, the EO chain had a little effect on the absorbance of reactive blue 19. However, the maximum absorbance intensity was increased and a shoulder was formed (Figure 8b) with the concentration of surfactant increased to 4.60 mmol/L (above CMC). The association between the absorbance of dye and the concentration of surfactant was well documented in the literature [18,34]. After the addition of nonionic surfactants with different EO chains in the reactive blue 19 solution, some changes were also observed in the spectrum of dye. There was a red shift in the UV-vis spectrum of reactive blue 19 because the shoulder peak was shifted from 617 to 628 nm. Furthermore, the absorbance intensity of shoulder peak decreased as the increase of EO chain.

It had been reported that strong hydrogen bond could be formed between anthraquinone structure and aqueous molecules [34]. The molecular rearrangement also existed as the resonance effects in aqueous solution [35,36,37]. When the EO chain was shorter in the molecular structure of surfactant, a stable interfacial membrane could be formed at the interface between water and siloxane [38]. And more of surfactants would adsorb to the surface of micelles, with the result that more reactive dyes were aggregated near micelles, which would form the intramolecular or intermolecular hydrogen bond between the dyes and the surfactants [19,39,40]. However, when the EO chain was increased in the molecular structure of nonionic surfactants, the solubility of surfactants in aqueous solution would increase. This would weaken the aggregation of dyes chromophores on micelles surface and have a slight increase in absorbance intensity.

**Figure 9:**
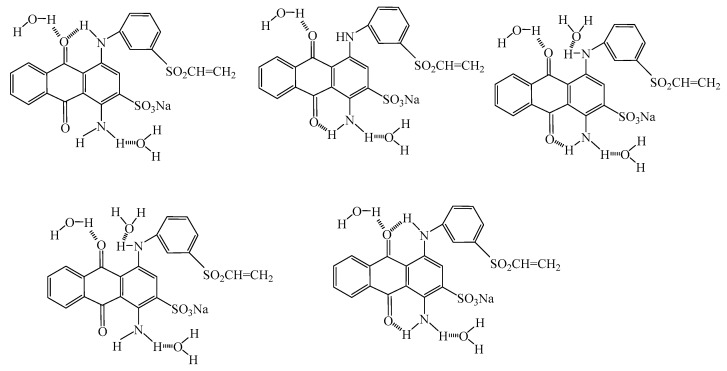
Possible structures of reactive blue 19 in aqueous solution.

From the above results and analysis, hydrogen bond and resonance effects are believed to result in changes in the UV spectrum. In alkyl alcohol polyoxyethylene ether aqueous solution, fatty alkyl chain could interact with the hydrophobic part of dye through the hydrophobic interaction. The polar amino group (–NH_2_) of dye can also form a intermolecular hydrogen bond with the EO group of surfactant. Some possible structures of reactive blue 19 in aqueous solution were shown in Figure 9.

## 4. Conclusions

In this paper, we have systemically studied the influence of ethylene oxide content on the hydrolysis of vinyl sulfone reactive dye in a siloxane reverse emulsion dyeing system. The HPLC results showed that the hydrolytic reaction of vinyl sulfone reactive dye was quick when there was no nonionic surfactant in the siloxane reverse emulsion dyeing system. However, the hydrolyic reaction was reduced when some alkyl alcohol polyoxyethylene ethers were added into the dyeing system. In particular, the EO chains in molecular structure of alkyl alcohol polyoxyethylene ether did influence the hydrolytic behavior of vinyl sulfone dye. The longer of EO chains, the faster the hydrolysis rate constant of vinyl sulfone dye. The changes of the absorption spectrum of dye underline that more of dyes were solubilized into the formed micelles and dye-surfactant complexes adhered to the surface of micelles when the EO chain was shorter, with the result that an intramolecular or intermolecular hydrogen bond was formed between dye and surfactant and the hydrolytic reaction of vinyl sulfone dye was slowdown. However, the aggregation of dye would be weakened when the EO chain was long. Meanwhile, solubility of surfactants in siloxane non-aqueous media would decrease and the hydrolytic reaction of vinyl sulfone dye was accelerated. As a result, some surfactants can be added to improve the fixation of reactive dye during the dyeing process. Furthermore, washing times after dyeing and the ecological problems can be decreased.

## Figures and Tables

**Table 1:** Gradient elution system for HPLC analysis.

Time (min)	Solvent A (%)	Solvent B (%)
0	61	39
20	60	40

**Table 2:** Overall reaction rate constants (*k*) for the vinyl sulfone dye and the squares of the correlation coefficients in linear regression (*R*^2^) at different ethylene oxide content in siloxane non-aqueous dyeing system.

Surfactant	Concentration (mmol/L)	*k*/min^−1^	*R* ^2^
Control	/	5.47 × 10^−2^	0.997
AEO-3	4.60	2.24 × 10^−2^	0.994
AEO-5	4.60	2.91 × 10^−2^	0.986
AEO-7	4.60	3.26 × 10^−2^	0.985
AEO-9	4.60	3.60 × 10^−2^	0.989
